# Sustained disease-free survival achieved with withdrawal of immunosuppression after rapid relapse of myelodysplastic syndrome following myeloablative allogeneic hematopoietic transplantation: a case report

**DOI:** 10.1186/1752-1947-7-18

**Published:** 2013-01-14

**Authors:** Betty K Hamilton, Gregory Vereb, Edward A Copelan

**Affiliations:** 1 Bone Marrow Transplant Program, Department of Hematologic Oncology and Blood Disorders, Cleveland Clinic Taussig Cancer Institute, 9500 Euclid Avenue, Cleveland, OH 44195, USA; 2 Wake Forest School of Medicine, Winston-Salem, NC, USA

## Abstract

**Introduction:**

Relapse after allogeneic hematopoietic stem cell transplantation in patients with myelodysplasia is a challenging problem with limited treatment options. Attempts to induce a graft-versus-leukemia effect have been used with limited success. In patients with myelodysplasia, sustained complete remissions have generally been limited to patients with long-term remission after transplant and those with low numbers of marrow blasts.

**Case presentation:**

We report the case of a 41-year-old Caucasian woman with relapsed myelodysplastic syndrome and a high blast percentage six months after undergoing an allogeneic transplant who achieved a sustained complete remission after withdrawal of immunosuppression alone.

**Conclusion:**

This case highlights the importance of a reasonable period of observation after withdrawing immunosuppression to induce graft-versus-leukemia, and the potential effectiveness of that approach.

## Introduction

Allogeneic hematopoietic stem cell transplantation (HSCT) is the only curative treatment for patients with myelodysplastic syndrome (MDS). Transplant-related complications and disease relapse are the major causes of treatment failure. Therapeutic options to manage disease relapse are limited and virtually all patients ultimately die of their disease or from complications of its treatment. Although withdrawal of immunosuppression is a common initial therapeutic strategy, its limited record of success commonly results in proceeding rapidly to induction chemotherapy and donor lymphocyte infusion (DLI) in patients with significant numbers of blasts. Chemotherapy, second transplants and DLI are often used but carry a high degree of morbidity and mortality. Here we present the case of a patient with relapsed MDS after allogeneic HSCT who was successfully treated with immunosuppression withdrawal.

## Case presentation

Our patient was a 41-year-old Caucasian woman who, four years prior to her presentation to the Cleveland Clinic, was found on routine blood work to be pancytopenic (white blood cell count (WBC) 3300/mm^3^ with an absolute neutrophil count (ANC) of 1600/mm^3^, hemoglobin 11.2g/dL, platelet count 104,000/mm^3^). She noted fatigue but denied other symptoms. An initial bone marrow biopsy showed trilineage dyspoiesis with an increase in atypical myeloblasts (8%), consistent with MDS, categorized as refractory anemia with excess blasts (RAEB-1). A cytogenetic analysis revealed a normal female karyotype. Her initial score on the International Prognostic Scoring System (IPSS) was 0.5, or intermediate-1 risk group. She did not require transfusions and her serum ferritin was 11.3ng/mL.

A subsequent bone marrow biopsy approximately six months later demonstrated a rising blast percentage to 15%, consistent with MDS RAEB-2. Our patient’s blood counts declined very gradually and remained largely stable over the next three years. The blast count on repeat bone marrows over this time period remained in the 10% to 15% range. Cytogenetic analysis continued to demonstrate a normal female karyotype. She remained asymptomatic and declined therapy, despite the elevated blast count. She began to develop worsening cytopenias despite a stable blast percentage on repeat bone marrow biopsies and she subsequently developed worsening anemia requiring red blood cell transfusions. As she became more symptomatic, she agreed to proceed with allogeneic HSCT from her brother, who had previously been identified to be human leukocyte antigen (HLA)-identical. A pretransplant bone marrow biopsy revealed high-grade MDS with 15% blasts. One month later, she underwent HSCT following high-dose intravenous busulfan (12.8mg/kg) and cyclophosphamide (120mg/kg). Graft-versus-host disease (GVHD) prophylaxis consisted of cyclosporine and mycophenolate mofetil.

Four weeks after the transplant she developed an erythematous, pruritic skin rash on her chest, accounting for approximately 25% of her body surface area. Results from a skin biopsy confirmed grade 2 acute GVHD. She also developed nausea and vomiting suggestive of upper gastrointestinal tract GVHD, although this was not confirmed on histology. She was treated with 1mg/kg of prednisone and continued mycophenolate and cyclosporine. Her GVHD symptoms resolved and she was tapered off prednisone by 18 weeks post-transplantation. Within this same time frame, it was first noted 125 days post-transplant that her blood counts had decreased (WBC, 1450/mm^3^; ANC, 870/mm^3^; hemoglobin, 9.7g/dL; platelet count, 83,000/mm^3^) although our patient felt well. An engraftment analysis from her peripheral blood on the same day, which had previously demonstrated complete donor leukocyte and T-cell chimerism, demonstrated 3% of the peripheral blood leukocyte deoxyribonucleic acid (DNA) of recipient origin and 41% of the T-cell-enriched fraction containing DNA of recipient origin. Her blood counts continued to decline. Sixteen days later, approximately five months after transplantation, her WBC was 940/mm^3^, ANC 430/mm^3^, hemoglobin 8.0g/dL and platelet count 89,000/mm^3^.

By day 143, a peripheral smear showed a WBC of 1240/mm^3^, ANC 520/mm^3^, hemoglobin 10.1g/dL, and a platelet count of 102,000/mm^3^ with 1% circulating blasts. A bone marrow biopsy at this time, now six months after transplantation, demonstrated relapsed MDS with 12% blasts. Cytogenetic analysis showed mixed chimerism but a normal karyotype, 46,XX/46,XY.

A repeat engraftment analysis on day 153 confirmed increasing recipient chimerism, with 42% of the peripheral blood leukocyte DNA of recipient origin and 65% of the T-cell-enriched fraction containing DNA of recipient origin. Peripheral blood counts on day 153 demonstrated a WBC of 1700/mm^3^, ANC 650/mm^3^, hemoglobin 9.3g/dL, and a platelet count of 124,000/mm^3^ with 10% circulating blasts. Our patient’s immunosuppression (cyclosporine and mycophenolate mofetil) was stopped day 153 and induction chemotherapy and DLI were planned.

Our patient was admitted to an outside hospital for fever and chest pain the following week. Blood counts at that time revealed a WBC of 8000/mm^3^ and an ANC of 1500/mm^3^ with no circulating blasts. She was found to have pneumonia with an associated parapneumonic pericardial effusion. She underwent pericardiocentesis and was transferred to our institution, where she completed a course of intravenous antibiotics. During her hospitalization, her WBC and ANC remained in the normal range with no evidence of circulating blasts. On follow-up, approximately a month after stopping immunosuppresion, our patient developed an erythematous, pruritic rash on her upper and lower extremities, accounting for approximately 50% of her body surface area. Counts at this time dropped, with a WBC of 98/mm^3^, hemoglobin 8.3g/dL, and a platelet count of 5000/mm^3^. A skin biopsy confirmed grade 2 GVHD. A bone marrow biopsy done the same day, five weeks after stopping immunosuppression, demonstrated a hypocellular bone marrow with no evidence of MDS. Engraftment analysis on day 181 demonstrated only 1% of peripheral blood leukocyte DNA of recipient origin and no recipient DNA in the T-cell enriched fraction.

The rash progressed over the next two weeks, becoming more erythematous and pruritic, and demonstrating features of both acute and chronic GVHD. Her liver function tests were also mildly elevated. Prednisone 1mg/kg was initiated and lead to a gradual improvement of her rash and liver function tests, and she was tapered off immunosuppression over a six-month period. Her blood counts normalized by day 209 and she continued to demonstrate complete donor chimerism. Interestingly, she developed intermittent neutropenia during her taper, however this would recover spontaneously and she continued to demonstrate complete donor chimerism on engraftment studies. Her counts have subsequently completely normalized. Our patient is currently more than three years from her allogeneic transplant and more than two and a half years from stopping immunosuppression. She continues to do exceedingly well, training for and running in half-marathons regularly. Her blood counts are normal and her leukocytes and T-cells are 100% donor.

## Discussion

The curative effect of HSCT results not only from the radiation and/or chemotherapy in the conditioning regimen, but also from a graft-versus-leukemia (GVL) effect exerted by the donor-derived immune system. The demonstration that donor T-cells inhibit the growth of leukemia colonies *in vitro* and eliminate leukemia stem cells, as assessed by a model of engraftment and propagation of leukemia in immunologically susceptible mice, indicate that this immunologic effect can be curative [1,2]. The lower incidence of relapse in patients who develop GVHD supports the contribution of the donor-derived immune system to the eradication of malignant cells [3]. The prevention or moderation of GVHD without loss of a GVL effect remains a significant and unsolved challenge.

Disease relapse is the most frequent cause of treatment failure after allogeneic HSCT of MDS. Relapse risk depends on remission status at the time of transplant, cytogenetic and molecular abnormalities of the MDS cells, and other factors. Patients with MDS who relapse after transplantation have dismal prognoses and there is no standard effective treatment strategy. Options include intensive chemotherapy and DLI, or second transplants, all of which are associated with high mortality rates and have achieved only very limited degrees of success [4]. Novel approaches to enhance the GVL effect using donor-derived natural killer cells are being studied [5].

Withdrawal of immunosuppression and DLI has been moderately successful in the treatment of relapsed chronic myelogenous leukemia (CML), which is highly sensitive to the GVL effect. High relapse rates after T-cell depletion of the allograft in CML transplantation has been well documented and support the sensitivity of CML to GVL [3]. Reports of individual patients who relapse after transplantation but achieve remission when immunosuppression was stopped and GVHD flared, and the effectiveness of DLI in achieving long-term remission in patients who have relapsed provide further evidence of this.

By contrast, patients with MDS and acute myelogenous leukemia (AML) who relapse after HSCT are a heterogeneous group and there is no single standard approach. Outcomes with aggressive chemotherapy, DLI or second HSCT are generally poor [6-8]. The few who benefit are generally younger patients with good performance status and long disease-free interval after transplant [4]. Immunosuppression withdrawal alone has rarely been reported to achieve a meaningful response after myeloablative transplantation in MDS or AML [9,10].

Researchers at the MD Anderson Cancer Center reported durable remissions in a small subset of patients with MDS or AML who had relapsed after reduced intensity, not myeloablative, regimens and undergone second HSCT, but dismal outcomes in patients who had their immunosuppression withdrawn or who received DLI [11]. Only 6.6% of patients achieved a complete remission after immunosuppression withdrawal; and unlike our patient, all had less than 10% blasts in their bone marrow and no circulating blasts. Most of those who did not respond to immunosuppression withdrawal were treated with further chemotherapy or DLI. Results after myeloablative transplant are even less encouraging [9,10].

A large series evaluating immunosuppression withdrawal demonstrated durable remissions in 84% of patients with CML, underscoring the strong GVL effect in CML, whereas only 10% of patients with AML and no advanced CML or acute lymphoblastic leukemia achieved remissions at three years [11]. Success was limited to patients who had sustained complete remission after transplant and who had low numbers of blasts. Patients with MDS who relapse shortly after myeloablative transplants with high numbers of blasts have not achieved sustained remission after withdrawal of immunosuppression alone and generally proceed rapidly to more aggressive therapy if appropriate.

The efficacy of DLI in MDS has been evaluated with a response rate of 14% to 40% [12,13]. DLI has been effective in inducing long-term remission in a small subset of patients with MDS; however, responses are associated with severe GVHD, which can result in significant transplant-related morbidity and mortality. The incidence rates of acute and chronic GVHD varied from 34% to 60% and 14% to 33%, respectively. In one small series, patients who were higher risk on the IPSS appeared to have a better response to DLI than those with lower-intermediate risk IPSS [13]. While low tumor burden generally is a predictor of response to DLI, MDS is a clinically and molecularly heterogeneous group, and one may speculate that the susceptibility to immunologic treatment or GVL effect may be impacted by the cytogenetic and molecular characteristics of the MDS clone.

The difficulty of determining who may be susceptible to immunologic treatment or the GVL effect was recently illustrated by a report of patients who relapsed after haploidentical transplantation [14]. In five of 17 patients’ mutant leukemic cells, the HLA haplotype that differed from the donor was lost at relapse due to acquired uniparental disomy of chromosome 6p. Thus, T-cells from the donor that had recognized and killed the original leukemic cells no longer recognized the mutant leukemic cells.

Other therapies, such as hypomethylating agents, with or without DLI or second transplants, also warrant further investigation in this setting. A recent report demonstrated the safety and efficacy of azacitidine as a treatment for minimal residual disease [15]. Twenty patients with decreasing donor chimerism were treated with four cycles of azacitidine and 80% of these patients responded with either increasing donor chimerism or stabilization in the absence of relapse. Although relapse eventually occurred in 65% of these patients, time to relapse was significantly delayed.

There remains significant variability in regards to long-term disease control in patients with MDS who relapse after transplant. Several factors are involved, including the disease status at time of transplant, remission duration, disease tempo at time of relapse, molecular and cytogenetic abnormalities, and donor type. The presence of comorbidities, including infections and active GVHD, also play an important role in determining subsequent therapy. Our case illustrates that immunosuppression withdrawal remains a valid and efficacious therapeutic modality in MDS relapse after allogeneic transplant. Although our patient had a higher blast percentage than has generally been associated with withdrawal of immunosuppression being effective, her disease was relatively slow in its evolution both initially and at relapse, and thus may have allowed for the adequate time required for immunosuppression withdrawal to work. Cytogenetic and molecular risk may have further played a role in disease kinetics as well as sensitivity of our patient’s MDS clone to immune manipulation.

## Conclusion

Although the approach to relapsed MDS or AML after allogeneic transplant remains challenging, our case highlights the importance of at least four weeks’ observation for GVHD or hematologic improvement following immunosuppression withdrawal. Even in patients who have been identified as having factors that would predict poor response to immunosuppression withdrawal, it should be considered as an initial tactic in cases of relapsed disease where an immediate response is not required. Unnecessary toxic chemotherapy and severe GVHD after DLI, both of which achieve only limited success, can be avoided with this approach.

## Consent

Written informed consent was obtained from the patient for publication of this manuscript. A copy of the written consent is available for review by the Editor-in-Chief of this journal.

## Abbreviations

AML: Acute myelogenous leukemia; ANC: Absolute neutrophil count; CML: Chronic myelogenous leukemia; DLI: Donor lymphocyte infusion; GVHD: Graft-versus-host disease; GVL: Graft-versus-leukemia; HLA: Human leukocyte antigen; HSCT: Hematopoietic stem cell transplantation; IPSS: International Prognostic Scoring System; MDS: Myelodysplastic syndrome; WBC: White blood cell count.

## Competing interests

The authors declare that they have no competing interests.

## Authors’ contributions

BKH analyzed and interpreted the patient data regarding the hematological disease and the transplant and was a major contributor to writing the manuscript. GV reviewed the pertinent literature and was a major contributor to writing the manuscript. EAC was a major contributor to writing the manuscript. All authors read and approved the final manuscript.
